# Synergy of Spin‐Orbit Torque and Built‐In Field in Magnetic Tunnel Junctions with Tilted Magnetic Anisotropy: Toward Tunable and Reliable Spintronic Neurons

**DOI:** 10.1002/advs.202203006

**Published:** 2022-08-04

**Authors:** Di Wang, Ziwei Wang, Nuo Xu, Long Liu, Huai Lin, Xuefeng Zhao, Sheng Jiang, Weinan Lin, Nan Gao, Ming Liu, Guozhong Xing

**Affiliations:** ^1^ Key Laboratory of Microelectronic Devices and Integrated Technology Institute of Microelectronics Chinese Academy of Sciences Beijing 100029 China; ^2^ School of Integrated Circuits University of Chinese Academy of Sciences Beijing 100049 China; ^3^ Department of Electrical Engineering and Computer Sciences University of California Berkeley CA 94720 USA; ^4^ School of Microelectronics University of Science and Technology of China Hefei 230026 China; ^5^ School of Microelectronics Northwestern Polytechnical University Xi'an 710072 China; ^6^ Department of Physics Xiamen University Xiamen 361005 China

**Keywords:** built‐in field, domain wall motion, leaky integrate‐and‐fire, spin‐orbit torque, spintronic neurons, tilted magnetic anisotropy, self‐reset

## Abstract

Owing to programmable nonlinear dynamics, magnetic domain wall (DW)‐based devices can be configured to function as spintronic neurons, promising to execute sophisticated tasks as a human brain. Developing energy‐efficient, CMOS compatible, reliable, and tunable spintronic neurons to emulate brain‐inspired processes has been a key research goal for decades. Here, a new type of DW device is reported with biological neuron characteristics driven by the synergistic interaction between spin‐orbit torque and built‐in field (*H*_built‐in_) in magnetic tunnel junctions, enabling time‐ and energy‐efficient leaky‐integrate‐and‐fire and self‐reset neuromorphic implementations. A tilted magnetic anisotropic free layer is proposed and further executed to mitigate the DW retrograde motion by suppressing the Walker breakdown. Complementary experiments and micromagnetic co‐simulation results show that the integrating/leaking time of the developed spintronic neuron can be tuned to 12/15 ns with an integrating power consumption of 65 µW, which is 36× and 1.84× time and energy efficient than the state‐of‐the‐art alternatives, respectively. Moreover, the spatial distribution of *H*_built‐in_ can be modulated by adjusting the width and compensation of the reference layer, facilitating tunable activation function generator exploration. Such architecture demonstrates great potential in both fundamental research and new trajectories of technology advancement for spintronic neuron hardware applications.

## Graphical abstract

A new type of domain wall‐based magnetic tunnel junction device is demonstrated with neuron characteristics driven by synergistic spin‐orbit torque and built‐in field, enabling biological leaky‐integrate‐and‐fire and self‐reset neuromorphic implementations. The integrating/leaking time can be tuned to 12/15 ns with an integrating power consumption of 65 µW, facilitating new applications of time‐ and energy‐efficient spintronic neuron hardware.

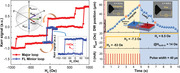

## 1 Introduction

Nonvolatile memories have attracted intensive attention in both research fields of cache and in‐memory computing, promising as building blocks for information storage and processing with high data transfer speed and low power consumption.^[^ ^1^, ^2^, ^3^, ^4^, ^5^ ^]^ Remarkably, novel nonvolatile neuromorphic devices, for example, resistive memristor,^[^ ^6^ ^]^ phase change memory,^[^ ^7^ ^]^ ferroelectric field‐effect transistor,^[^ ^8^ ^]^ and spintronic memory^[^ ^9^ ^]^ have recently demonstrated the great potential to alleviate the memory wall bottleneck in traditional von Neumann architecture. Among them, owing to the intriguing physical properties and nonlinear dynamics, the spintronic devices arouse extraordinary research and application prospects for neuromorphic computing that emulate specific biological characteristics in a full‐time‐, area‐, and energy‐efficient way.^[^ ^9^ ^]^ Recently, there are some hardware explorations for spintronic neuron applications.^[^ ^10^, ^11^, ^12^ ^]^ However, their development is still in the early stage of research, especially for the neuron devices based on magnetic domain wall (DW) motion driven by spin‐orbit torque (SOT) with merits of high speed, low power consumption, and outstanding endurance.^[^ ^2^, ^13^, ^14^, ^15^ ^]^

Moreover, promoted as one of the most applicable neuron models, the leaky‐integrate‐and‐fire (LIF) model has been widely used to guide the construction of neuron devices.^[^ ^16^ ^]^ Some spintronic structural concepts have been proposed with micromagnetic simulations, for example, embedded hard ferromagnet constructed DW LIF neuron^[^ ^17^ ^]^, shape anisotropy and magnetocrystalline anisotropy gradient‐based DW/skyrmion LIF neurons.^[^ ^18^, ^19^, ^20^, ^21^ ^]^ On the one hand, the aforementioned spintronic neuron devices require an unavoidable peripheral circuit to realize complete neural functions with additional area and energy overhead. On the other hand, the motive force of magnetic solitons relied on the spin‐transfer torque (STT) in all the above devices, which in turn bear high power costs and severe reliability issues due to prerequisite high injected current. This is because of the limited spin‐polarized current results from low spin polarization of the ferromagnetic layer,^[^ ^22^ ^]^ the serious shunting, and interfacial scattering effect.^[^ ^23^ ^]^ In contrast, SOT driven magnetic texture evolution mechanism demonstrates superior virtues with high energy efficiency and operation speed.^[^ ^2^ ^]^ Recently, one of the typical SOT‐based LIF neuron devices utilizes the thermal effect between two continuous current pulses to assist Hall bar probabilistic switching to simulate LIF characteristics.^[^ ^24^ ^]^ Despite this scheme being feasible, a large amount of heat accumulation and thermal sensitivity hinders the on‐chip integration and large‐scale application. In contrast, devices based on magnetic solitons (e.g., DW, skyrmion) are expected to develop toward the nanometer scale, and their combination with SOT is expected to develop high‐speed and high‐density devices. Compared to skyrmion, that is, difficult to be created, manipulated, and read out,^[^ ^25^ ^]^ magnetic DWs‐based neuromorphic nanodevices are amenable to be realized experimentally. Nevertheless, a nanoscale single SOT‐DW‐based LIF neuron device with membrane capacitor free, self‐reset, and high spiking frequency is highly desirable in CMOS compatible integration way.

In the present work, we experimentally demonstrated the feasibility of synergistic interaction between SOT‐driven DW motion and built‐in field (*H*_built‐in_) of a reference layer (RL) as driving and leaking motive to mimic the LIF and self‐reset characteristics of biological neurons. Furthermore, our study reveals that when the leaking field is larger than the Walker breakdown field, the chiral precession of DW leads to stochastic motion of instantaneous SOT driving DW, resulting in retrograde motion with device LIF‐reset misoperations. On this basis, we designed the free layer (FL) with characteristics of tilted magnetic anisotropy (TMA). Extensive simulation results prove that the TMA can suppress the chiral precession of DW effectively and play a critical role in modulating DW motion velocity. Meanwhile, such LIF‐reset neuron devices with different firing rates and operation frequency characteristics can also be tuned by adjusting the local width and compensation of RL.

## 2 Results and Discussion

### 2.1 Properties and Characterization of the Film Stack

**Figure 1:**
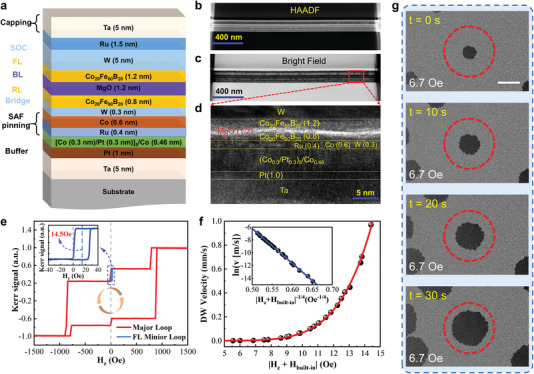
Basic structure and magnetic characteristics of specific film samples for neuron devices. a) Schematic of films stack sequence (thickness not in scale). Corresponding images of b) HAADF and c) STEM. d) The enlarged cross‐section image of HRTEM. e) Major and minor K‐H loops of the films stack sample. f) DW motion velocity as a function of field and g) corresponding Kerr images. The scale bar is 500 µm.

A series of film stack samples were deposited by magnetron sputtering, consisting of substrate/Ta(5)/Pt(1)/[Co(0.3)/Pt(0.3)]_5_/Co(0.46)/Ru(0.4)/Co(0.6)/W(0.3)/CoFeB(0.8)/MgO(1.2)/CoFeB(1.2)/W(5)/Ru(1.5)/Ta(5), the units of the thickness in parentheses are nanometers, as shown in **Figure** **1**a. The high‐angle annular dark‐field (HAADF) and high‐resolution transmission electron microscopy (HRTEM) images are shown in Figures 1b and 1c, respectively, demonstrating the high quality and smooth film stack. The enlarged cross‐section image of HRTEM shown in Figure 1d also illustrates a great agreement with the high interface quality of the sample among different layers. A polar magneto‐optic Kerr effect (p‐MOKE) microscope was used to measure the hysteresis loop, indicating the good perpendicular magnetic anisotropy (PMA) and built‐in field of 14 Oe according to the shift of the minor loop, as shown in Figure 1e. To explore the DW dynamics, the dependence of DW velocity on the field was calculated through continuous snapshotting of Kerr images in different fields as depicted in Figure 1f, which satisfies the creep law of DW.^[^ ^26^ ^]^ The typical Kerr images of the domain are shown in Figure 1g.

### 2.2 Demonstration of LIF Characteristics Based on PMA FL

**Figure 2:**
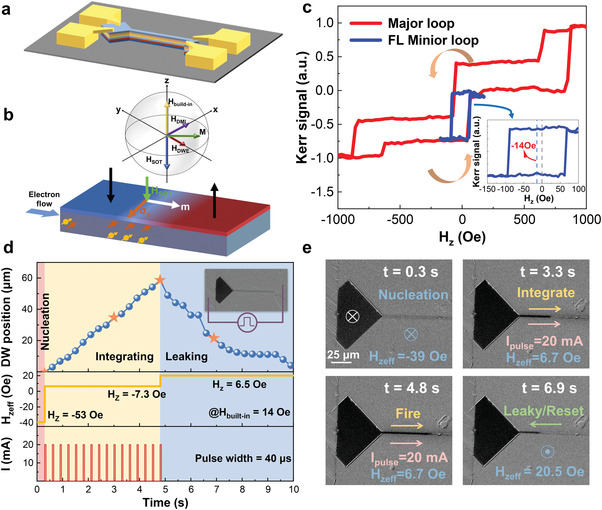
Spintronic spiking neuron device and corresponding magnetic and dynamic LIF processes with self‐reset properties. a) Schematic of the patterned device structure. b) Principle of SOT and the various effective fields acting on DW. c) Major and minor K‐H loops of the device. d) DW position, effective *H*_z_, and current sequence as a function of time. e) Corresponding MOKE images at different times and marked with star‐like symbols in (d).

**Figure** **2**a demonstrates the stripe‐like device via patterning through Argon ion beam etching, followed by deposition of 40 nm thick SiO_2_ encapsulation isolation and Ti (20 nm)/Au (80 nm) electrodes by sputtering and thermal evaporation, respectively. The DW motion was driven by the SOT mechanism as schematically shown in Figure 2b. Briefly, the spin polarization *σ* originated from the spin Hall effect (SHE) along the *y*‐axis, so that the effective field *m* × *σ* induced by SOT is negative with a left‐hand DW configuration. Other effective fields acting on DW, for example, shape anisotropy induced DW energy field (*H*_DWE_), Dzyaloshinskii–Moriya interaction (DMI) field (*H*_DMI_), and built‐in field (*H*_built‐in_) from RL^[^ ^27^, ^28^ ^]^ are illustrated in the inset of Figure 2b. As depicted in Figure 2c, both major and minor loops measured from devices’ hysteresis curves indicate a good PMA and the shift of the minor loop implies a 14 Oe *H*_built‐in_ stemmed from RL. Clearly, the SOT effective field competes with the *H*_built‐in_ as designed. When the applied current density is large enough, SOT can drive DW to move rightward, while DW moves toward the left upon synthetic *H*_built‐in_ without any extra current application. Evidently, the LIF characteristics of neurons were emulated in a biological manner with tunable DW velocity driven by SOT.

Specifically, a pulsed nucleation *H*_z_ field of −53 Oe was applied first to inject a DW and a sequence of current of 20 mA with 40 µs width was then injected from the left terminal under an out‐of‐plane (OOP) *H*_z_ field of −7.3 Oe. The phenomenon of DW moving rightward could be observed because SOT was large enough to overcome the effective built‐in field (*H*_built‐in_ + *H*_z_ = 6.7 Oe) and DW depinning field.^[^ ^29^, ^30^ ^]^ After DW reaches the threshold site under the effect of sequential 16 current pulses, an OOP field *H*_z_ of 6.5 Oe was applied with the effective *H*_built‐in_ as 20.5 Oe, so that the reset‐like leaking process of DW can be clearly observed. A complete LIF process of DW with the application of corresponding net/effective *H*_built‐in_ and current is shown in Figure 2d. In the case of positive effective *H*_built‐in_, the down‐up DW motion followed the same direction as the charge current, excluding the driving force of either thermally activated field or STT,^[^ ^31^ ^]^ validating the dominating role of the SOT effect.^[^ ^2^ ^]^ Note that the nonuniform velocity of DW and the slightly pinning phenomena during the leaking process are attributed to the random pinning sites introduced during film depositions and device fabrications.^[^ ^30^ ^]^ Figure 2e shows the measured MOKE images during the LIF process marked in Figure 2d with star‐like symbols. The detailed DW LIF process can be found in Video S1 and Figure S1, Supporting Information.

### 2.3 Retrograde DW Motion in PMA LIF Neuron

**Figure 3:**
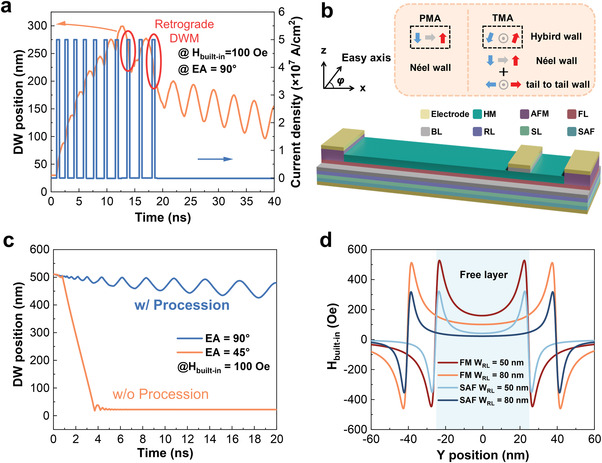
DW dynamics under SOT‐driven DWM during LIF process and proposed TMA device. a) Retrograde DWM during the LIF process through micromagnetic simulations. b) Schematic of proposed TMA‐device structure and a sketch of mechanism. c) Suppression of TMA on DW precession. d) Spatial distribution of *H*_built‐in_ of single ferromagnetic layer and SAF layer along the width direction.

The DW motion observed from experiments generally falls into the creep, depinning, or flow regimes.^[^ ^26^ ^]^ In line with the original intention of high‐speed applications, the DW motion with a faster speed in the device is expected at a higher field. However, the Walker breakdown field (*H*_WB_) of most common ferromagnetic materials is usually only tens or hundreds of Oe.^[^ ^28^, ^32^, ^33^, ^34^ ^]^ When *H*_built‐in_ is larger than *H*_WB_, the DW velocity will drop rapidly and enter the precession regime due to the periodic variation of DW chirality.^[^ ^33^, ^35^ ^]^ If a DW moves into the precession regime during the leaking process, the instantaneous direction of DW motion will depend on the synergistic effect of SOT and DW chirality when the state of DW transits from leaking to integrating. Consequently, the motion direction of DW will behave in a stochastic manner, leading to potentially unexpected misoperation in neuron device execution, as shown in **Figure** **3**a. The parameters used in the micromagnetic simulator MuMax3^[^ ^36^ ^]^ are shown in Table S1, Supporting Information. To calculate the dynamics of DW, the Landau–Lifshitz–Gilbert equation was employed with an additional SOT term which can be given as^[^ ^2^, ^37^ ^]^

(1) $$\begin{array}{c} \frac{dm}{dt}=-\gamma m\times H_{\mathrm{eff}}+\alpha m\times \frac{dm}{dt}+\gamma H_{\mathrm{DL}}m\times \left(m\times \sigma \right)+\gamma H_{\mathrm{FL}}m\times \sigma \end{array}$$

where m, *γ*, *H*_eff_, *α*, and *σ* refer to unit magnetization, gyromagnetic ratio, effective field, Gilbert damping factor, and direction of spin polarization, respectively. The four terms on the right side of the equation describe the field torque, Gilbert damping torque, damping‐like SOT, and field‐like SOT, respectively. *H*_DL_ = ℏ*θ*_SH_ *J*/(2*eM*_s_ *t*_F_) and *H*_FL_ = *βH*_DL_ define the strength of damping‐like SOT and field‐like SOT, where ℏ, *e, θ*_SH_ *, J, M*_s_, *t*_F_, and *β* refer to the reduced Planck's constant, electron charge, spin Hall angle, charge current density, saturation magnetization, the thickness of FL, and the ratio of the field‐like SOT to damping‐like SOT, respectively. *H*_eff_ mainly consists of the terms^[^ ^37^ ^]^

(2) $$\begin{array}{c} H_{\mathrm{eff}}=\frac{2A_{\mathrm{ex}}}{M_{s}}\frac{\partial^{2}m}{\partial x^{2}}+\frac{2K}{M_{s}}m_{z}\hat{z}+\frac{2K_{d}}{M_{s}}m_{y}\hat{y}-\frac{2D_{0}}{M_{s}}\left(\hat{y}\times \frac{\partial m}{\partial x}\right)+H_{\mathrm{stray}} \end{array}$$

where *A*_ex_, *K*_d_, and *D*_0_ refer to the exchange stiffness constant, hard‐axis anisotropy, and DMI constant, respectively.

### 2.4 Proposal of TMA LIF Neuron and Suppression of Walker Breakdown

To alleviate the problem of misoperation, we proposed an FL (i.e., DW motion conduit) with characteristic TMA in MTJ device, as shown in Figure 3b, which can be obtained through a dedicated oblique angle sputtering approach,^[^ ^38^ ^]^ interlayer exchange coupling,^[^ ^39^ ^]^ exchange spring,^[^ ^40^ ^]^ and/or thermal annealing.^[^ ^41^ ^]^ The tilted angle of the easy axis (EA) is defined by *φ*, expressed as [cos(*πφ*/180°), 0, sin(*πφ*/180°)]. In the FL with TMA, the anisotropy field lies in the *x*–*z* plane, that is, the FL has both PMA and IMA so that the DW is composed of Néel/intermediate wall (for PMA) and tail‐to‐tail/head‐to‐head wall (for IMA), as shown in the inset of Figure 3b. As a result, *H*_WB_ will be determined jointly by IMA and PMA. *H*_WB_ can be increased and the precession of DW can be suppressed due to the larger hard‐axis anisotropy of IMA compared to that of PMA.^[^ ^42^ ^]^ Dean et al.^[^ ^43^ ^]^ also reported that *H*_WB_ will increase as EA decreases when EA is greater than 60°. As indicated in Figure 3c, DW move without any precession in the case of EA with 45°, in contrast to DW motion accompanied by obvious precession in a PMA film, which shows a good agreement with the above inference. Compared to other methods used to suppress Walker breakdown through the emission of spin waves, for example, periodic width structure^[^ ^44^ ^]^ and periodic holes,^[^ ^45^ ^]^ the scheme of TMA is more suitable for devices based on DW propagation. Besides, *H*_WB_ can also be suppressed by increasing DMI, because *H*_WB_ will be dominated by larger DMI (*H*_WB_ ≈ *αH*_DMI_).^[^ ^28^, ^34^ ^]^ Noted that the SOT‐based DW LIF neuron device is expected to work in the steady regime to avoid retrograde DW motion‐induced stochastic misoperation. Moreover, as shown in Figure 3d, the leaking field (*H*_built‐in_) of RL can be precisely modulated through size effect and field strength compensation^[^ ^46^ ^]^ by manipulating the spatial distribution of *H*_built‐in_ (Figure S2, Supporting Information). Furthermore, various types and characteristics of neuron devices and activation function generators, for example, the sigmoid function generator, can be realized by modulating the local width of RL (Figure S3, Supporting Information).

### 2.5 Basic and LIF Characteristics of TMA LIF Neuron

**Figure 4:**
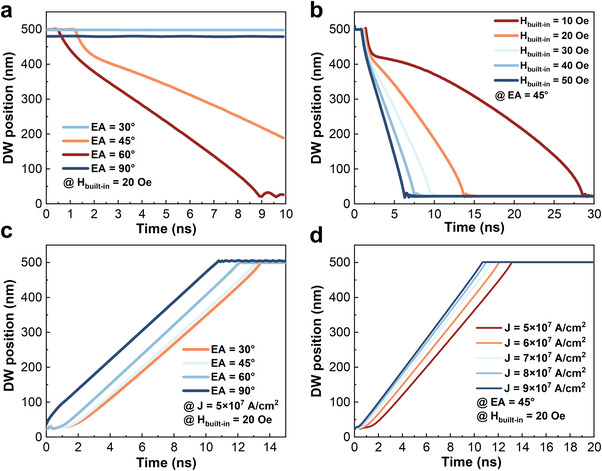
Modulation of DW velocity during integration and leaking processes. DW velocity as a function of a) the easy axis and b) *H*_built‐in_ during the leaking process. DW velocity as a function of c) easy axis and d) current density during the integration process.

**Figure 5:**
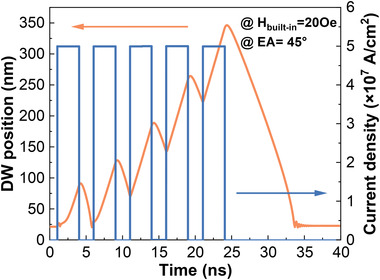
Dynamic LIF process with self‐reset features of proposed spintronic neuron devices.

**Figure** **4** illustrates the DW integrating and leaking process for different EA and driving forces. As shown in Figure 4a, the velocity of DW during the leaking process reaches a maximum in the case of EA at 60°, in contrast to DW without depinning in the PMA film. However, it is found that when EA continues to decrease (i.e., toward in‐plane), the velocity of DW decreases rapidly. This is because the tail‐to‐tail wall component of the hybrid DW becomes more dominant as EA decreases under an OOP *H*_built‐in_, resulting in lower DW velocity. Figure 4b shows the velocity of DW increases as *H*_built‐in_ increases during the leaking process. While in the integrating process, the velocity of DW does not change significantly with the change of EA, as shown in Figure 4c. This may be due to the internal conversion of velocity during the transformation between the Néel wall and tail‐to‐tail wall, both of which can be driven by SOT in the presence of DMI.^[^ ^47^ ^]^ Figure 4d shows that the velocity of DW increases as current density increases during the integrating process. The whole LIF process of DW is demonstrated in **Figure** **5** and Video S2, Supporting Information, with consolidated integrating and leaking times of ≈12 and ≈15 ns, respectively, which is 7.4× and 36× times more efficient than other DW LIF‐^[^ ^17^, ^18^, ^21^ ^]^ and traditional CMOS LIF‐neuron devices,^[^ ^48^ ^]^ respectively. The power consumption of the neuron device is estimated at about 65 µW during the integrating process, which is 1.84× lower than that of other DW LIF neuron devices.^[^ ^17^ ^]^ It is foreseeable that time and energy efficiency can be further improved by scaling down and utilizing the topological insulators and metal alloys with large spin Hall angles (e.g., BiSb, BiSe, and PtAu).^[^ ^2^ ^]^

## 3 Conclusion

In summary, based on experimentally corroborated prototype domain wall devices, we demonstrated a new type of reliable single neuron cell with LIF and self‐reset functionalities for spintronic neuron applications based on magnetic tunnel junction, implementing CMOS compatible, energy‐efficient, and tunable neuromorphic computing. The synergistic utilization of SOT and *H*_built‐in_ with interfacial DMI at interfaces between the ferromagnetic free layer and heavy metal provides an effective magnetic domain wall motion and pinning manipulation, facilitating LIF and self‐reset iterations under full electric‐field control. To the best of our knowledge, the proposed design realizes all LIF and self‐reset functions with a significantly reduced energy consumption and complexity of device units and peripheral circuits. This enables fan‐out and cascading functions and promises great potential for futuristic reconfigurable and high‐speed spintronic neuron applications, for instance, the voice recognition for embedded AIoT and neuromorphic computing hardware acceleration.

## 4 Experimental Section

### Film Preparation and Characterization

The films consisting of substrate/Ta(5)/Pt(1)/[Co(0.3)/Pt(0.3)]_5_/Co(0.46)/Ru(0.4)/Co(0.6)/W(0.3)/CoFeB(0.8)/MgO(1.2)/CoFeB(1.2)/W(5)/Ru(1.5)/Ta(5) were deposited at room temperature onto high‐resistivity Si/SiO_2_ wafers by DC and RF magnetron sputtering. Base pressure of ≤1 × 10^−6^ Pa and Ar gas were used for the sputtering. HRTEM and HAADF‐STEM were used to characterize the structure of the cross‐sectional.

### Device Fabrication and Measurement

The films were fabricated into strip devices of 2 µm width and different lengths by optical lithography and ion beam etching. The MOKE microscopy with a MagVision system was used to scan the hysteresis loop of the film and take images of domain wall motion. Both the time interval and displacement of the domain walls were extracted from the MOKE images and used to calculate the DW velocity.

## Conflict of Interest

The authors declare no conflict of interest.

## Supporting information

Supporting Information

Supplemental Video 1

Supplemental Video 2

## Data Availability

The data that support the findings of this study are available from the corresponding author upon reasonable request.
