# Supplementary material for: Synergy of Spin‐Orbit Torque and Built‐In Field in Magnetic Tunnel Junctions with Tilted Magnetic Anisotropy: Toward Tunable and Reliable Spintronic Neurons
Source: Adv Sci (Weinh). 2022 Aug 4;9(30):2203006. doi: 10.1002/advs.202203006 (PMC9596820; doi:10.1002/advs.202203006)
Supplement: Supplementary file 1 — Supporting Information [file ADVS-9-2203006-s001.pdf]

ADVANCED SCIENCE
Open Access

Supporting Information

for *Adv. Sci.*, DOI 10.1002/advs.202203006

Synergy of Spin-Orbit Torque and Built-In Field in Magnetic Tunnel Junctions with Tilted Magnetic Anisotropy: Toward Tunable and Reliable Spintronic Neurons

*Di Wang, Ziwei Wang, Nuo Xu, Long Liu, Huai Lin, Xuefeng Zhao, Sheng Jiang, Weinan Lin, Nan Gao, Ming Liu and Guozhong Xing**

Synergy of spin-orbit torque and built-in field in magnetic tunnel junctions with tilted magnetic anisotropy: towards tunable and reliable spintronic neurons

Di Wang[1,2,a)], Ziwei Wang[1,2,a)], Nuo Xu[3], Long Liu[1,2], Huai Lin[1,2], Xuefeng Zhao[1,4], Sheng Jiang[5], Weinan Lin[6], Nan Gao[4], Ming Liu[1,2] and Guozhong Xing[1,2,*]

[1]Key Laboratory of Microelectronic Devices & Integrated Technology, Institute of Microelectronics, Chinese Academy of Sciences, Beijing, 100029, China
[2]University of Chinese Academy of Sciences, Beijing, 100049, China
[3]Department of Electrical Engineering and Computer Sciences, University of California, Berkeley, CA, 94720 USA
[4]School of Microelectronics, University of Science and Technology of China, Hefei, 230026, China
[5]School of Microelectronics, Northwestern Polytechnical University, Xi'an 710072, China
[6]Department of Physics, Xiamen University, Xiamen 361005, China

[a)]These authors contributed this work equally.
[*]Author to whom correspondence should be addressed: gzxing@ime.ac.cn (G.Z. Xing)

Table of Contents

Figure S1 Velocity of DW as a function of current density.

Table S1 Mumax3 simulation parameter.

Figure S2 Spatial distribution of stray field of 80 nm width SAF RL.

Figure S3 Proposed sigmoid activation function generator.

Movie S1 Whole LIF process of DW in the experiment.

Movie S2 Whole LIF process of proposed TMA neuron device.

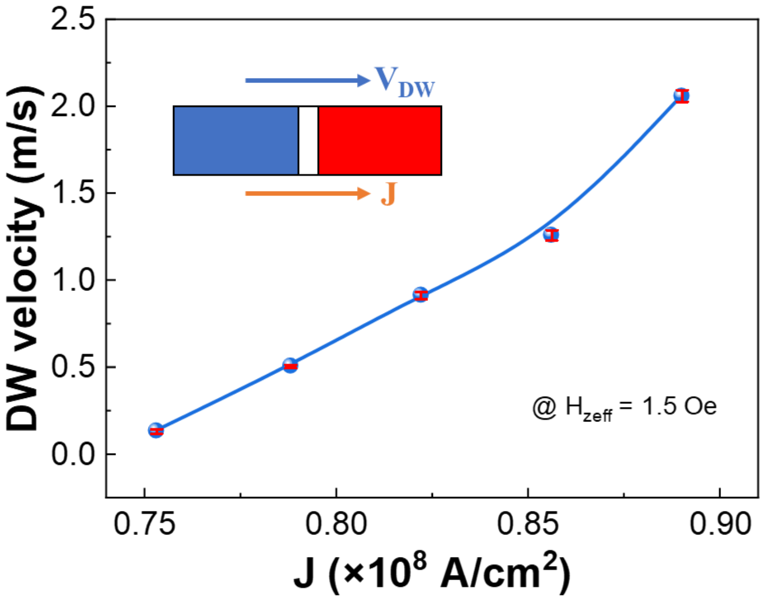

Figure S1 Velocity of DW as a function of current density.

Table S1 MuMax3 simulation parameters.

Parameter	Description	Values
**M_s**	**Saturation magnetization**	**1×10^6 A/m**
**K_u**	**Uniaxial magnetic anisotropy**	**8×10^5 J/m^3**
**A**	**Exchange stiffness constant**	**1×10^{-11} J/m**
**α**	**Gilbert damping constant**	**0.02**
**DMI**	**Dzyaloshinskii-Moriya interaction strength**	**0.2 mJ/m^2**
**β**	**FLT/DLT ratio**	**1**
**θ**	**Spin hall angle**	**-0.3**
**H_{stray}**	**Stray field**	**20 Oe to 100 Oe**
**$(L\times W)_{AFM}$**	**Boundary antiferromagnetic pinning layer dimension**	**10×50 nm^3**
**$(L\times W\times t)_{FL}$**	**Free layer dimension**	**520×50×1 nm^3**

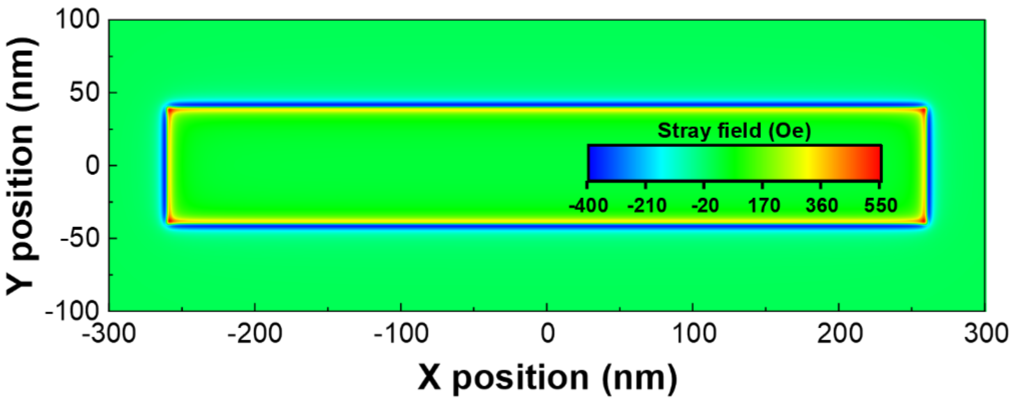

Figure S2 Simulated spatial distribution of stray field from 80 nm wide SAF RL.

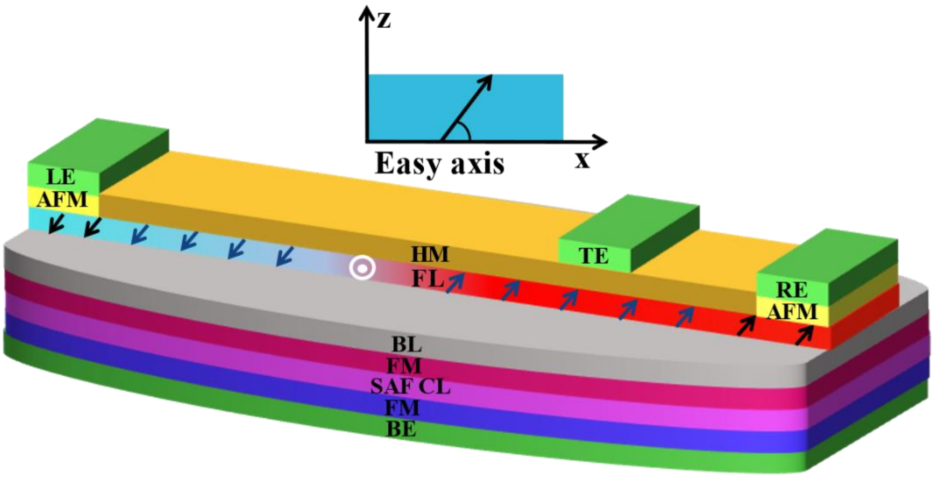

Figure S3 Proposed Sigmoid activation function generator.
